# Chemotherapy in Retinoblastoma: Current Approaches

**DOI:** 10.4274/tjo.06888

**Published:** 2015-12-05

**Authors:** Özge Yanık, Kaan Gündüz, Kıvılcım Yavuz, Nurdan Taçyıldız, Emel Ünal

**Affiliations:** 1 Ankara University Faculty of Medicine, Department of Ophthalmology, Ankara, Turkey; 2 Ankara University Faculty of Medicine, Department of Radiology, Ankara, Turkey; 3 Ankara University Faculty of Medicine, Department of Pediatrics, Ankara, Turkey

**Keywords:** Retinoblastoma, chemoreduction, intra-arterial chemotherapy, intravitreal chemotherapy, subconjunctival chemotherapy

## Abstract

Retinoblastoma (RB) is the most common childhood malignant intraocular tumor. Although enucleation and external beam radiotherapy have been historically used, today the most commonly used eye-sparing approach is chemotherapy. Chemotherapy can be used in both intraocular and extraocular RB cases. Chemotherapeutic agents may be applied in different ways, including systemic, subconjunctival, intra-arterial and intravitreal routes. The main purposes of application of systemic therapy are to reduce the tumor size for local treatment (chemoreduction), or to reduce the risk of metastasis after enucleation surgery (adjuvant therapy). Intra-arterial chemotherapy with the current name “super-selective intra-arterial infusion therapy” could be applied as primary therapy in tumors confined to the retina or as a secondary method in tumor recurrence. The most important advantage of intra-arterial therapy is the prevention of systemic chemotherapy complications. Intravitreal chemotherapy is administered in the presence of persistent or recurrent vitreous seeding. The term “extraocular RB” includes orbital invasion and metastatic disease. Current treatment for orbital invasion is neoadjuvant chemotherapy followed by surgical enucleation and adjuvant chemotherapy and radiotherapy after surgery. In metastatic disease, regional lymph node involvement, distant metastases, and/or central nervous system (CNS) involvement may occur. Among them, CNS involvement has the worst prognosis, remaining at almost 100% mortality. In metastatic disease, high-dose salvage chemotherapy and autologous hematopoietic stem cell rescue therapy are the possible treatment options; radiotherapy could also be added to the protocol according to the side of involvement.

## INTRODUCTION

Retinoblastoma (RB) is the most common malignant intraocular tumor in children.^1^ It accounts for approximately 4% of all pediatric malignancies,^2^ occurring in one in every 15,000-20,000 live births.^3^ The disease can be sporadic or familial, bilateral or unilateral, hereditary or non-hereditary, and is classified accordingly in three distinct groups. Involvement is unilateral in two thirds of cases and bilateral in one third; 6% of cases have a positive family history.^2^

The tumor currently known as ‘RB’ was first described by Petras Pawius in 1597.^4^ Enucleation was first recommended for treating RB in 1809 by James Wardrop, an ophthalmologist from Edinburgh,^5^ after which this treatment modality was used for nearly two centuries. Originally called Fungus haematodes, the tumor was first named RB by Verhoeff and has been referred to by that name since 1926.^5^

The tumor-suppressing RB gene, located on the long arm of the chromosome 13, is responsible for the pathogenesis of RB.^6^ Knudson et al.’s^7^ two-hit hypothesis elucidated tumor pathogenesis. Mutations in both alleles of the gene are necessary for tumor development. Non-hereditary RB patients need to acquire mutations in both alleles, while hereditary RB patients have an inherited mutation in one of the alleles, so only one acquired mutation may result in RB. For this reason, the average age at diagnosis is lower in hereditary RB patients.

**Figure 1:**
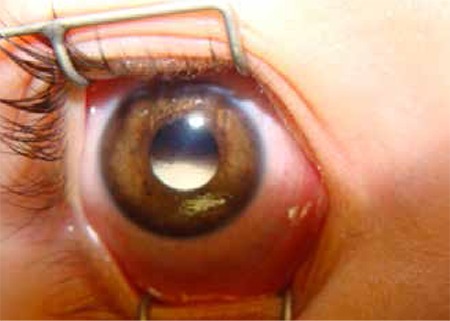
Leukocoria in retinoblastoma

RB is diagnosed at an average age of 18 months, with 24 months for unilateral cases and 12 months for bilateral cases.^8^ Leukocoria is the most common finding at presentation (Figure 1).^9^ Leukocoria is the appearance of a white reflection through the pupil. Other common symptoms are strabismus and vision loss. Neovascularization, neovascular glaucoma, hyphema, pseudohypopyon and vitreous hemorrhage may be observed in advanced cases. Proptosis may occur in cases with extraocular extension or as a result of orbital cellulitis. Unlike many tumors, RB can be diagnosed based on clinical findings alone, without the need for biopsy.

The first classification system for RB, the Reese-Ellsworth classification system, was developed in 1969. The system is based on tumor number, size and position, and essentially evaluates the potential success of external beam radiotherapy. With the development of other treatment options like chemotherapy, this classification system lost importance. Currently in use is the International Classification of RB (ICRB) (Table 1).^10^ This classification is based on chemoreduction success and provides more information about globe prognosis than systemic prognosis. The currently accepted grading system for RB is the International RB Grading System (Table 2),11 which allows for the evaluation of both intraocular and extraocular tumors.

### Retinoblastoma Treatment

**Figure 2:**
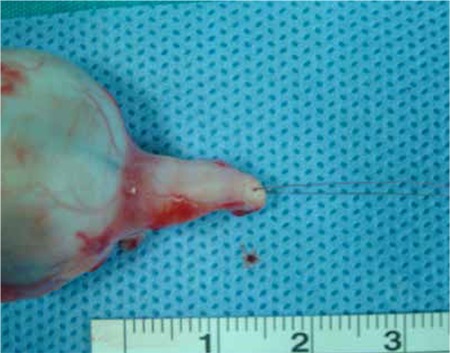
A long (~15 mm) optic nerve resection in enucleation. Due to retinoblastoma extension into the central nervous system through the optic nerve, a long resection can be life-saving

Treatment of RB requires a multidisciplinary approach. Ophthalmologists, pediatric oncologists, radiation oncologists and neurointerventional radiologists work as a team in the treatment process. Basic treatment methods are enucleation, external beam radiotherapy, chemotherapy, transpupillary thermotherapy, plaque radiotherapy and cryotherapy. Enucleation and external beam radiotherapy have been used as primary treatment methods for approximately a century (Figure 2). Chemotherapy methods are a commonly used approach in current clinical practice. The main objectives of RB treatment are firstly patient survival, then protection of the eye and finally visual function. For this reason, there are cases still treated with primary enucleation.

### Chemotherapy

Chemotherapy is used in the treatment of both intraocular and extraocular RB, and its administration may be systemic, subconjunctival, intra-arterial or intravitreal. One of the main reasons for using systemic chemotherapy for RB is to avoid complications related to radiation and the development of secondary cancers.

### Chemotherapy in Intraocular Retinoblastoma

#### 1. Systemic Chemotherapy

Systemic chemotherapy may be administered for chemoreduction or as an adjuvant therapy. It allows the management of intraocular disease and lowers risk of metastasis. In addition, the administration of systemic chemotherapy has been reported to prevent pineoblastoma and secondary cancers.^12,13^

**Figure 3:**
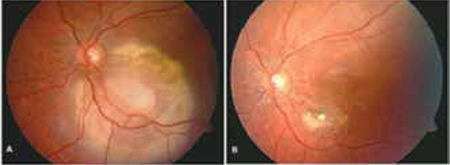
A) Macular retinoblastoma before chemoreduction. B) Tumor focus shows complete regression after 6 cycles of chemoreduction

**Figure 4:**
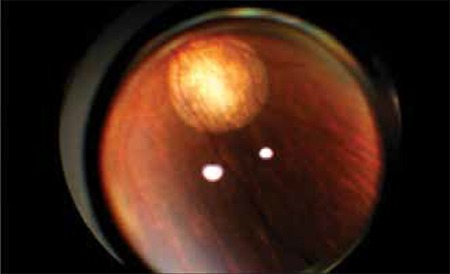
Partial regression after chemoreduction and type IV chorioretinal scar due to cyrotherapy

**Figure 5:**
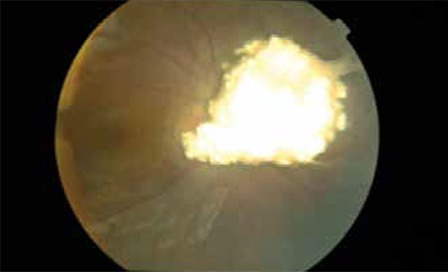
Regressed calcific retinoblastoma focus (type I regression pattern) after chemoreduction

a) Chemoreduction: Chemoreduction is used in the treatment of intraocular RB. The aim is to shrink the tumor in order to facilitate local treatment methods. Rarely (10%), chemoreduction alone can be sufficient (Figure 3), but 90% of cases require local treatment methods in combination (Figure 4). It is used in unilateral and bilateral cases.^14^ The main effect of chemoreduction occurs after two cycles; average reductions of 35% in tumor base diameter and 50% of tumor thickness can be achieved.^15^ Tumor regression may follow different regression patterns (Figure 5).^16^

Due to their good intraocular penetration, the standard chemotherapeutic agents used are vincristine, etoposide, and carboplatin (VEC protocol). These are administered for at least six cycles at 28 day intervals. The treatment protocol is shown in Table 3.^17^ After two cycles of chemotherapy and the tumor size and subretinal fluid have been reduced, local treatment methods are used. The most frequently used local treatment methods are cryotherapy, transpupillary thermotherapy and plaque radiotherapy. Shields et al.^10^ performed chemoreduction followed by local consolidation therapy in 249 eyes with group A, B, C or D RB. Treatment success was defined as the avoidance of external beam radiotherapy or enucleation; success rates of 100% in group A, 93% in group B, 90% in group C and 47% in group D were achieved. The most important problems that may arise after chemoreduction are tumor unresponsiveness and tumor recurrence. In a 2004 study by Gunduz et al.^18^ including 105 RB patients, unresponsive disease was observed in 9.5% and recurrence in 44.2% of the eyes. In 2013, a study from the same center including 171 eyes reported that 17.5% had persistent disease and 25.7% had tumor recurrence. Of these 171 eyes that initially underwent chemoreduction, 33.9% received external beam radiotherapy, 33.3% underwent secondary enucleation, and 4.1% received intra-arterial chemotherapy.^19^ In that study, tumor group (group D and E), presence of vitreous and subretinal seeds, tumor base width and thickness, and number of chemotherapy and local treatments administered were determined as risk factors for treatment failure (i.e. enucleation).

**Figure 6:**
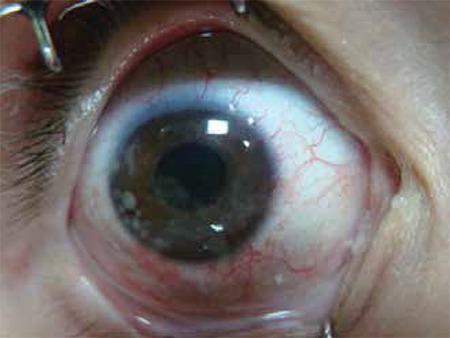
Anterior segment seeds found at initial presentation in a case with group E retinoblastoma according to the International Retinoblastoma Classification System

b) Adjuvant systemic chemotherapy: Adjuvant systemic chemotherapy is used after enucleation in patients with high-risk histopathological features such as invasion of the anterior chamber (Figure 6), optic nerve and choroid (Table 4).^20^ Kaliki et al.^21^ analyzed 509 eyes with group D and E tumors that were primarily enucleated and found histopathologic evidence of high metastasis risk in 17% and 24%, respectively; those cases were treated using the VEC protocol. Chemotherapy was not administered to patients in that study who did not have histological high-risk features, and none developed metastasis. In a study by Honavar et al.^22^ adjuvant treatment was administered after enucleation to 80 patients with histopathologic high-risk characteristics; 24% of the patients who did not receive adjuvant treatment developed metastases compared to only 4% of the patients who received adjuvant treatment. In another study by Kaliki et al.^23^ including 52 eyes, the VEC protocol was administered to patients with histopathologic high-risk features after enucleation and none developed metastases during the 66-month follow-up period.

Side effects related to systemic chemotherapy include bone marrow suppression, alopecia, autotoxicity and nephrotoxicity. Cases of acute myeloid leukemia due to etoposide have been reported, although it was specified that those patients received high doses of etoposide.^24^

#### 2. Subconjunctival Chemotherapy

**Figure 7:**
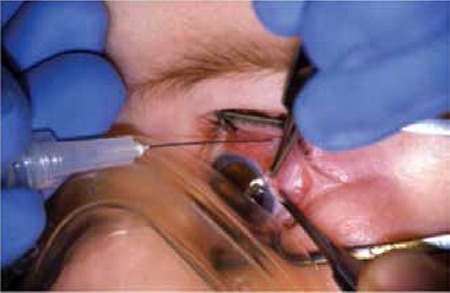
Subconjunctival carboplatin administration

Subconjunctival chemotherapy is not used alone, but is administered in conjunction with systemic chemoreduction in eyes with advanced stages of disease (group D and E) in order to increase the intraocular concentration of chemotherapeutic agents. Subconjunctival carboplatin is used at a dose of 10-20 mg (Figure 7). It may be administered after two cycles of chemoreduction up to three times with one month intervals.^25^Vitreous concentration 30 minutes after administration is 10 fold greater than with systemic delivery.^26^

The biggest limitation of subconjunctival chemotherapy is ineffective control of subretinal tumor seeding. Side effects include periorbital edema and cellulitis, orbital adipose tissue atrophy, fibrosis of extraocular muscles and Tenon’s capsule, and subsequent limitations to ocular motility.^27^

#### 3. Intra-arterial Chemotherapy

For RB, the term “intra-arterial chemotherapy” was first used approximately 60 years ago by Reese et al.^28^ to describe triethylenemelamine (TEM) injection into the internal carotid artery. Selective ophthalmic artery infusion was first described by Japanese investigators Yamane et al.^29^ A temporary occlusion was produced in the internal carotid artery distal to the orifice of the ophthalmic artery using a micro-balloon catheter, and chemotherapeutic agents were infused directly into the artery. For many years this method was not used in the western world. It was later popularized by Abramson et al.^30^ and modified to allow ophthalmic artery microcatheterization. The method currently in use was popularized by Abramson et al.^30^ and is known as superselective ophthalmic artery infusion.

**Figure 8:**
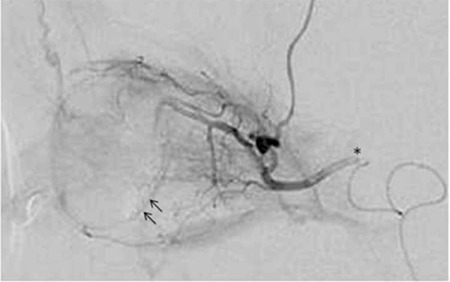
Checking retinal staining (arrows) by the injection of radio-opaque material through a microcatheter placed in the ostium of the ophthalmic artery (*) prior to intra-arterial chemotherapy administration

Superselective intra-arterial infusion is administered under fluoroscope over 30 minutes as pulsatile infusion (1 ml/min) via a microcatheter (400-570 µ) into the ostium region of the ophthalmic artery (Figure 8). In cases of ophthalmic artery vasospasm or occlusion or in patients with anatomic variations, if retinal staining can not be achieved through the ophthalmic artery, retinal staining through the middle meningeal artery via the external carotid artery is tried and intra-arterial chemotherapy is administered through this route.^31^ The main advantage of this procedure is the reduction of side effects and long-term complications related to systemic chemotherapy. The most commonly used chemotherapeutic agent is melphalan. Originally, melphalan dose was 3 mg, 5 mg or 7.5 mg based on the patient’s age and weight. In recent publications, however, it has been determined that a standard dose of 5 mg is necessary, and even a standard dose of 7.5 mg can be used.^32,33^ The total treatment dose should not surpass 15 mg/m2 in patients weighing over 10 kg, and 0.5 mg/kg in patients weighing 10 kg or less. Topotecan (1 mg) and carboplatin (30-50 mg) are alternative chemotherapeutic agents. Topotecan and melphalan can be used in combination, especially in the presence of dense vitreous seeds. Synergistic pharmacokinetic activity has been reported following combined administration.^34^

Superselective intra-arterial infusion is usually used as the primary treatment modality in sporadic and unilateral RB patients older than 4 months, and can be used as a secondary modality in cases with recurrent RB and recurrent subretinal or vitreous seeds despite systemic chemotherapy or plaque radiotherapy.^14^ It may also be used as a primary treatment in bilateral RB cases. After one application, mean reductions of 33% in tumor base circumference and 46% in tumor thickness were reported, and accompanying serous retinal detachment resolved at a rate of 76%.35 Standard administration is once a month, with a total of three cycles. The procedure is usually conducted under general anesthesia.

Gobin et al.^36^ treated 95 eyes of 78 patients with intra-arterial melphalan, topotecan, carboplatin or methotrexate as a primary or secondary treatment modality; they reported globe conservation at rates of 81.7% and 58.4% with primary and secondary treatment, respectively. Neutropenia was observed as a side effect at a rate of 11.4%. Shields et al.^37^ reported 72% globe conservation with primary treatment in 70 eyes of 67 patients (100% in group B and C tumors, 94% in group D tumors, and 36% in group E tumors). They reported 62% globe conservation with secondary treatment. Solid tumor regression was achieved in 94% of eyes and regression of subretinal and vitreous seeds was achieved in 95% and 87%, respectively.

Another advantage of intra-arterial chemotherapy is that it can hinder the development of new tumor foci in patients with genetic RB. Abramson et al.^38^ treated genetic RB patients with intra-arterial melphalan and observed new tumor foci development in 2.4% of the primary-treated group and 8.0% of the secondary-treated group.

General and ocular complications may arise as side effects of treatment. As the procedure is performed under general anesthesia, the most common complications to arise are anesthesia-related, such as bronchospasm.^36^ Theoretically, complications like groin hematoma, femoral artery occlusion, limb ischemia, stroke, seizure and neurological deficits could also occur, but if heparinization is maintained at the proper dose and proper technique is used, these complications are uncommon.^37^ Neutropenia is the most common hematological side effect.^36^ Administration through the ophthalmic artery results in the passage of chemotherapeutic agents to other ocular and orbital arteries including the supraorbital artery, posterior and anterior ciliary arteries, which can cause transient side effects in the forehead area such as hyperemia, eyelid edema, ptosis and chemosis. Furthermore, there have been reports of post-treatment vitreous hemorrhage, retinal artery occlusion, ophthalmic artery spasm and obstruction, partial choroid ischemia and optic neuropathy. Another unexpected complication that may arise following intra-arterial chemotherapy is the presence of intraocular foreign material. Eagle et al.^39^ investigated histological complications in 8 eyes that were enucleated for various reasons following intra-arterial chemotherapy and found foreign material with birefringence index in the blood vessels of 5 of the eyes. It is believed that this material may be cotton fibers from the operating table that contaminated the catheter tip. Another complication reported in the same study was the presence of retinal and choroidal thrombosis, which was attributed to drug toxicity.

#### 4. Intravitreal Chemotherapy

Because the vitreous is an avascular tissue and the systemic or intra-arterial administration of chemotherapy has limited transition to the vitreous, vitreous seeding is an especially important problem. The primary indication for intravitreal chemotherapy is persistent or recurrent vitreous seeds.^14^ Melphalan at a dose of 20-30 μg/0.1 ml is used as the chemotherapeutic agent. Serious complications like hypotonia and phthisis bulbi have been reported with melphalan doses over 50 μg.^40^ In early studies, administration was conducted once a month, but the current approach is once a week or in alternate weeks, with at least 6 intravitreal injections.

**Figure 9:**
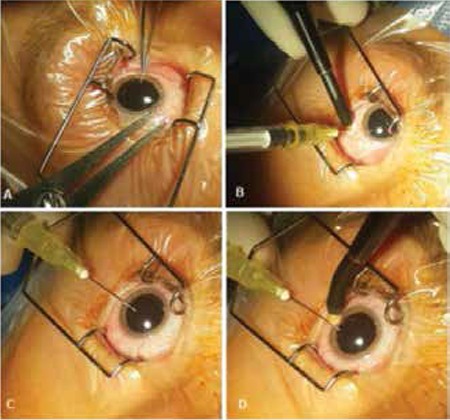
Intravitreal melphalan injection. (A) The pars plana area was marked with a gauge (3 mm from the limbus in accordance with the patient’s age); (B) after intravitreal injection, freeze-empty cryotherapy was applied three times during withdrawal of the 30-G injector; (C) paracentesis was performed due to elevated intraocular pressure; (D) freeze-empty cryotherapy was applied three times to the limbus during withdrawal of the paracentesis injector

Intravitreal chemotherapy is administered via 30-G injector in the pars plana region 1-2 clock hours from the vitreous seeds. To prevent the possible spread of the tumor, injection site cryotherapy can be performed 3 times while the injector is positioned in the eye for the first application (Figure 9). Afterwards, the melphalan can be dispersed through the vitreous by moving the eye left, right, up and down.

In a study of 11 patients with vitreous seeds in two or more quadrants who were treated with intravitreal melphalan, an average of 5 injections were performed and complete regression of vitreous seeds was observed in all the patients. No complications were observed aside from non-axial cataract in two patients and retinal pigment changes at the injection site in two patients.^41^ In another study including group D and E RB cases, 9 eyes were treated with 40 μg/0.04 ml melphalan and 8-20 μg/0.04 ml topotecan due to recurrent retinal seeds, and tumor control was achieved with an average of 1.9 injections of this dual therapy.^42^ However, enucleation was later performed in 33% of the patients due to persistent anterior chamber lesions and tumor recurrence.

The greatest concern regarding intravitreal chemotherapy is extraocular tumor extension and metastasis risk. In 1985, Karcioglu et al.^43^ performed fine needle aspiration biopsy in 4 enucleated eyes with intraocular tumor (3 of which were RB) and found tumor cells in 55% of the 11 needle tracks. As a result, intravitreal chemotherapy application was avoided for many years. In 2014, however, Smith et al.^44^ conducted a broad meta-analysis of complications related to 1,304 intravitreal injections performed in 315 eyes with RB between the years 1946 and 2013 and reported only 1 case of extraocular tumor extension. This indicates a 0.07% risk of extraocular tumor spread. In another study of 7 eyes that were enucleated for various reasons after intravitreal melphalan therapy, histopathological analysis revealed no evidence of tumor spread in any of the needle tracks.^45^

### Chemotherapy in Extraocular Retinoblastoma

**Figure 10:**
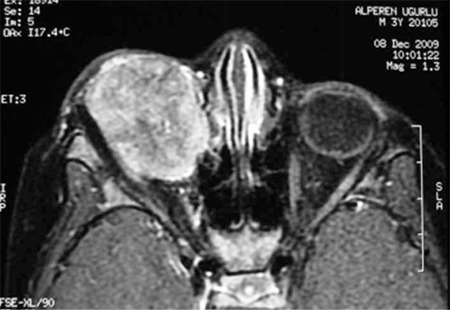
T1-weighted orbital magnetic resonance imaging of retinoblastoma-related buphthalmic bulbus oculi and orbital invasion

In developed countries, expected survival for intraocular RB patients with early diagnosis and treatment is over 95%; however, this rate is still in the 50% range worldwide.^2^ The primary cause of mortality in RB is extraocular extension.^46^ Extraocular extension includes orbital invasion (Figure 10), spread to local lymph nodes, distant metastasis and central nervous system (CNS) involvement.

In epidemiologic studies, especially in developing countries, the reported incidence of orbital RB is over 50%.47 Orbital invasion increases the risk of metastatic disease 10 to 27 fold compared with intraocular cases.48 Primary exenteration surgery is no longer necessary in the treatment of orbital RB due to the sensitivity of the tumor to chemotherapy. The current treatment approach is reducing the tumor mass with neoadjuvant chemotherapy, thereby avoiding exenteration, followed by enucleation and subsequent adjuvant chemotherapy and external beam radiotherapy to the orbit.^48^ The key point here is to exclude distant metastases and CNS involvement using a full diagnostic approach prior to treatment. Desired reduction of the orbital mass can be achieved over 3-6 cycles of neoadjuvant therapy, after which enucleation can be performed. Adjuvant chemotherapy is initiated two weeks after enucleation and continues for an average of 9 cycles. In addition, a 40 Gy dose of orbital radiotherapy is administered within 2 months following enucleation. After the exclusion of orbital involvement, CNS involvement and distant metastases, a high-dose VEC protocol can be applied (Table 5).^49^ Radhakrishnan et al.^49^ applied this treatment paradigm to 28 orbital RB patients and reported a 40.4% survival rate over a follow-up period of 14.75 months; they also reported that neoadjuvant therapy prevented exenteration surgery.

In RB, metastasis development usually occurs within the first year after diagnosis. Cases with distant metastases but without CNS involvement are stage IVa. In these cases, the distant metastases generally occur in the bone and bone marrow.^50^ Although high-dose chemotherapy has been beneficial in the treatment of metastatic RB, there is currently no standard chemotherapy protocol. A promising approach is induction chemotherapy (vincristine, cyclophosphamide, cisplatin, etoposide), which should be followed by autologous hematopoietic stem cell collection and high-dose chemotherapy (carboplatin, thiotepa, etoposide, topotecan). Additionally, radiotherapy can be applied to the region of involvement. Dunkel et al.^51^ applied this treatment regimen to 15 cases with distant metastasis and reported 67% survival at 5 years. In a similar study, 11 patients with metastatic disease were treated with high-dose chemotherapy and autologous hematopoietic stem cell rescue therapy, with disease-free survival reported in 7 patients after 39 months.^52^ Four patients with CNS involvement died due to recurrence occurring an average of 7 months later.

Of RB metastases, CNS involvement is the most common, leads to relapse most frequently, and has the worst prognosis, with nearly 100% mortality.^46,50^ Spread to the CNS usually occurs through the optic nerve, though hematogenous spread is also possible. High-dose chemotherapy and autologous hematopoietic stem cell rescue therapy are promising in the treatment of CNS involvement. This treatment can be combined with intrathecal chemotherapy and craniospinal radiotherapy. It may be an important approach in the prevention of CNS recurrences due to the high CNS penetration of thiotepa, a part of the treatment protocol. Dunkel et al.^53^ performed induction therapy followed by autologous hematopoietic stem cell collection and then high-dose chemotherapy in 8 patients with CNS involvement. In addition, craniospinal radiotherapy was performed in two patients and one patient was administered intrathecal anti-GD2 monoclonal antibody. With these treatments extended survival of 2 of the patients (40 months and 101 months) was achieved.

Clinical studies of chemotherapy in RB are ongoing. One of these is ARET0321, a study being conducted by Dunkel et al.^53^ aiming to standardize the combined application of chemotherapy, autologous stem cell transplantation and/or radiotherapy in patients with extraocular RB. Patient recruitment began in February 2008 and completion of the study is planned for February 2017. Another clinical study (NCT00445965) of iodine 131 monoclonal antibody 3F8 treatment of CNS and leptomeningeal tumors includes RB patients with CNS involvement. In the near future of RB chemotherapy, many novel molecules may be identified.

## CONCLUSION

In recent years, many new chemotherapy options have been identified for the management of RB and high success rates have been reported with these methods. However, despite current treatment approaches, RB continues to be a life-threatening pediatric disease, especially in cases with extraocular spread. Many critical factors including patient age, tumor location, size, and extension, and visual expectations, must be considered together when choosing treatment modalities. As the most important factor influencing treatment success is disease grade, early diagnosis remains critical in the treatment of RB.

## Figures and Tables

**Table 1:**
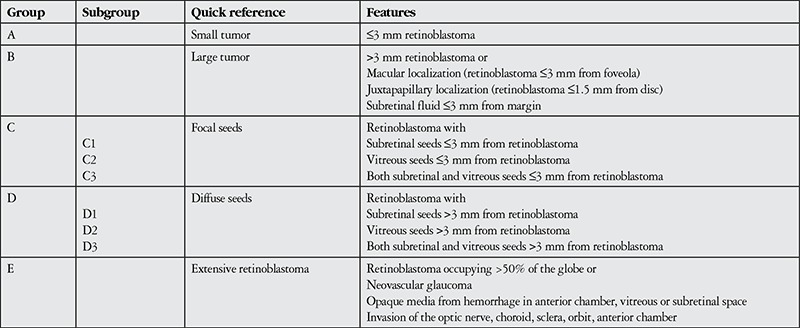
The international classification of retinoblastoma

**Table 2:**
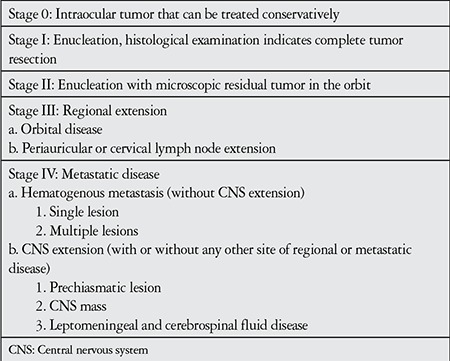
International retinoblastoma staging system

**Table 3:**
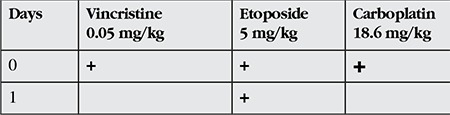
Standard VEC protocol

**Table 4:**
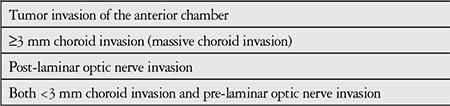
Histopathological high-risk factors for metastasis

**Table 5:**
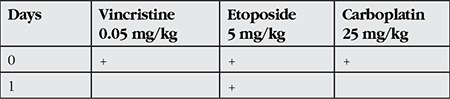
High-dose VEC protocol
